# Nanobiotechnology: A smart platform of the future transform liquid biopsy era

**DOI:** 10.1016/j.jlb.2024.100137

**Published:** 2024-01-03

**Authors:** Srijan Goswami, Palas Samanta, Manab Deb Adhikari

**Affiliations:** a Department of Biotechnology, University of North Bengal, Darjeeling, West Bengal, India; b Department of Environmental Science, Sukanta Mahavidyalaya, University of North Bengal, Dhupguri, West Bengal, India

**Keywords:** Cancer, Nanotechnology, Extracellular vesicles, Biomarkers, Sensor

## Abstract

Cancer is the leading cause of death worldwide. The most complicated fact about this disease is early diagnosis. Treatment at advanced stages of the disease is extremely challenging. We need an early cancer detection method. Nanotechnology-based liquid biopsy is an efficient platform for this health crisis. Several advanced modifications to this method ushered in a new era of liquid biopsy. The present article addresses the transforming signature of nanobiotechnology on liquid biopsy techniques (it explores its advantages and limitations).

Inventing a “simple and non-invasive” blood test for screening and diagnosis of cancer associated biomarkers has been a fundamental goal in oncology research and treatment. Analysing body fluids (for example: Blood [1], saliva [2], sweat [3], cerebrospinal fluid, amniotic fluid, semen, sputum, etc.) for circulating cancer biomarkers using Liquid Biopsy Technology is transforming the way clinical oncology is practiced [4]. Body fluids for liquid biopsies are composed of various types of biomarkers like Circulating Tumour DNA (CtDNA), Circulating Tumour Cells (CTC), Cell-Free DNA (cfDNA) [5], Extracellular Vesicles (EV) [6], Circulating RNA, Proteins and Metabolites each having their own significance. The journey of liquid biopsy method development described in Fig. 1.

**Fig. 1:**
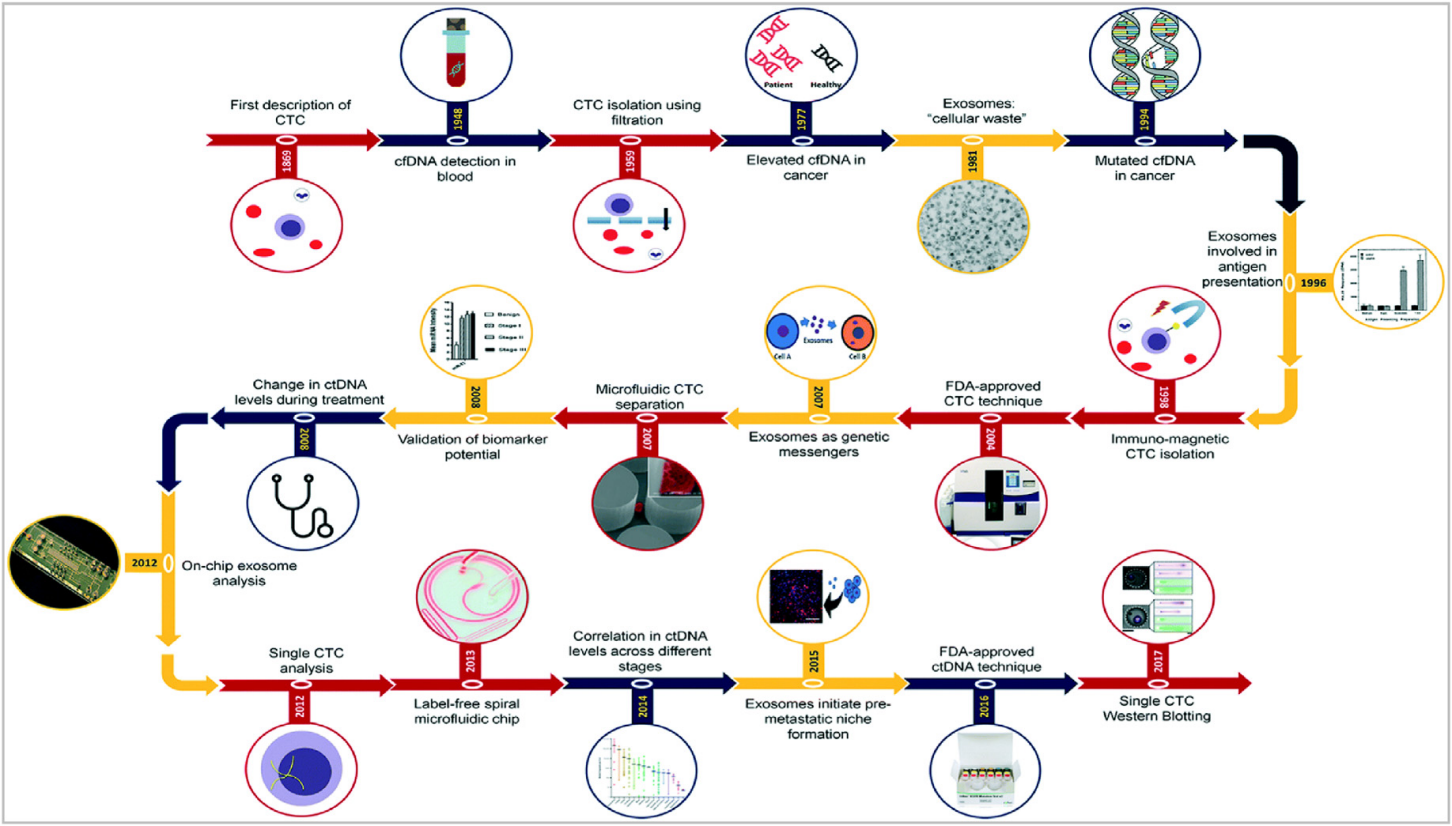
**Landscape of the journey of the liquid biopsy method.** (Reproduced with permission from ref. 33 Copyright 2018 Royal Society of Chemistry).

Current cancer research identifies an exciting component in human body fluid, which is called extracellular vesicles (EVs). This is a cellular messenger (it plays a vital role in cell-to-cell communication and the presence cell's status). Exosome is a subpopulation of EVs, it regulates complex cellular communication during cancer progression [7,8]. The most exciting fact about exosomes, they regulate tumour cell metastasis, cancer stem cell development, and drug and therapeutic resistance [7,9,10]. Exosomes-based clinical signature is described in Fig. 2.

**Fig. 2:**
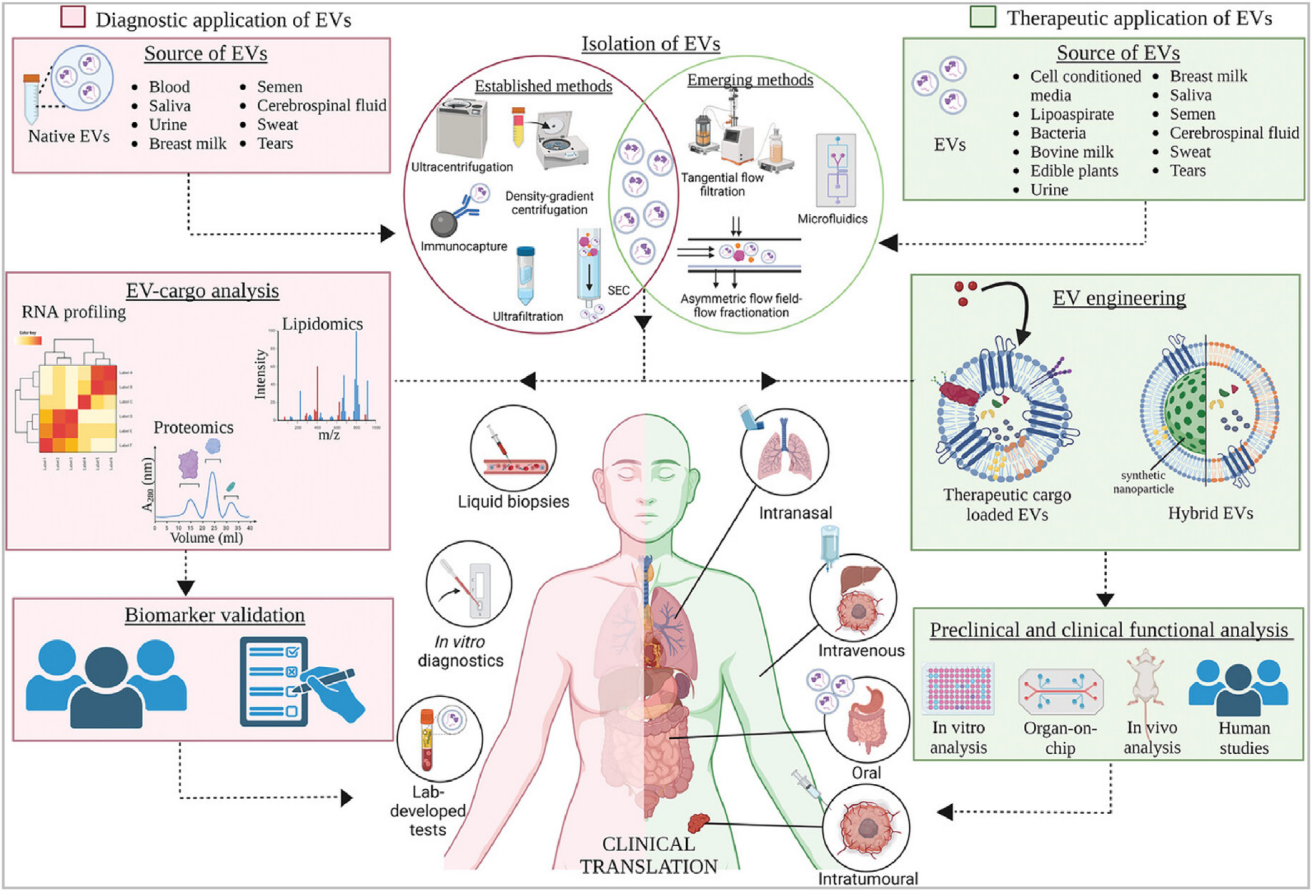
**Impact of exosome in clinical research.** (Reproduced with permission under Creative Commons CC BY 4.0 license from ref. 34 Copyright 2023 The Authors).

Exosome molecular cargo is efficient for biomarker research. An important aspect of Liquid Biopsy research is the development of novel nanobiotechnology-based analytical techniques for circulating exosomes (this platform-based research develops a solution for exosome heterogeneity-single exosome profiling, exosome barcoding) [[11], [12], [13], [14]]. Differential ultracentrifugation is used to separate exosomes from blood plasma, more precisely microfluidic device, Nano-electrochemistry-based sensor, and surface plasma resonance-based exosomes scanning is the landscape of impact of nanotechnology development in liquid biopsy [[15], [16], [17], [18]]. The complex contaminants in body fluids, as well as wide variations in surface biomarker types, are significant impediments to efficient exosome isolation and sensitive molecular quantification, as well as the further development and clinical application of exosome-based liquid biopsy. In recent years nanotechnology-based systems are widely being implemented in exosome biosensing and analysis leading to their effective purification and molecular studies [19]. Nanomaterials are widely implemented in liquid biopsy studies for improving biomarker detection sensitivity, specificity, and efficiency (see Fig. 3). They are essential in many aspects of liquid biopsy, ranging from sample preparation and isolation of circulating biomarkers to detection and analysis [19,20]. Gold Nanoparticles coupled with antibodies and nucleic acid probes capture and detect specific biomarkers like exosomes. They're used in detection techniques like surface-enhanced Raman spectroscopy (SERS) and colorimetric assays. Magnetic Nanoparticle-based separation techniques are being used to isolate and enrich circulating biomarkers such as CTCs or extracellular vesicles. They allow for quick sample preparation and biomarker purification. Quantum dots are semiconductor nanoparticles that have distinct optical properties. They are used as fluorescent labels for biomarkers in liquid biopsy assay, providing high sensitivity and multiplexing capabilities. Liposomes and exosome-mimicking nanoparticles are lipid-based nanoparticles that mimic or encapsulate extracellular vesicles. They are required for extracellular vesicle investigation and isolation for biomarker analysis [[20], [21], [22], [23]]. Nanobiotechnology enables the evolution of ultrasensitive and precise nanoscale sensors and probes. These can detect and quantify low levels of circulating biomarkers such as circulating tumour DNA (ctDNA), circulating tumour cells (CTCs), and extracellular vesicles in bodily fluids. Magnetic and functionalized nanoparticles are being implemented for efficient isolation and enrichment of circulating biomarkers. This reduces background noise and improves sample purity for downstream analysis. Multiplexed analysis in liquid biopsy is possible with nanomaterials such as quantum dots and nanoparticles with various surface modifications, allowing the detection of multiple biomarkers in a single sample [[24], [25], [26]]. Real-time progression of disease and response to treatment can efficiently be monitored using nanoscale sensors as they are suitable for point of care settings. This continuous data can help clinicians make more informed clinical decisions and adjust treatment plans as needed. Nanotechnology allows for the creation of miniaturized diagnostic devices, such as lab-on-a-chip systems, capable of processing and analysing liquid biopsy samples in compact and efficient manner. These portable devices can be used in point-of-care settings. Nanobiotechnology-assisted liquid biopsies can provide valuable genetic and molecular information to guide personalized treatment strategies. It enables real-time monitoring of genetic mutations and alterations, allowing clinicians to make more informed decisions. The biomarkers found in liquid biopsy can be coated using nanomaterial-based substances that may be utilized for enhancing their stability. Using nanomaterial-based coatings on fragile biomarkers in liquid biopsy helps preserve and maintain the integrity of the sample during transport and storage. Conventional biopsies are a time-consuming and highly sophisticated process and thus require expensive apparatus, infrastructure, and skilled professionals, and all these requirements make it expensive. On the contrary liquid biopsy processes are simple and non-invasive (or minimally invasive) thus making these processes less expensive as compared to conventional biopsy. Nanotechnology-based liquid biopsy detects genetic mutations and other cancer-related changes in circulating tumour DNA or CTCs, allowing for early cancer diagnosis. This is especially beneficial for cancers that are difficult to detect through traditional methods. Liquid biopsy is a new field in clinical oncology, and the use of nanobiotechnology increases the possibility of new biomarkers and innovative detection methods, resulting in an evolution of diagnostic strategies [[24], [25], [26]].

**Fig. 3:**
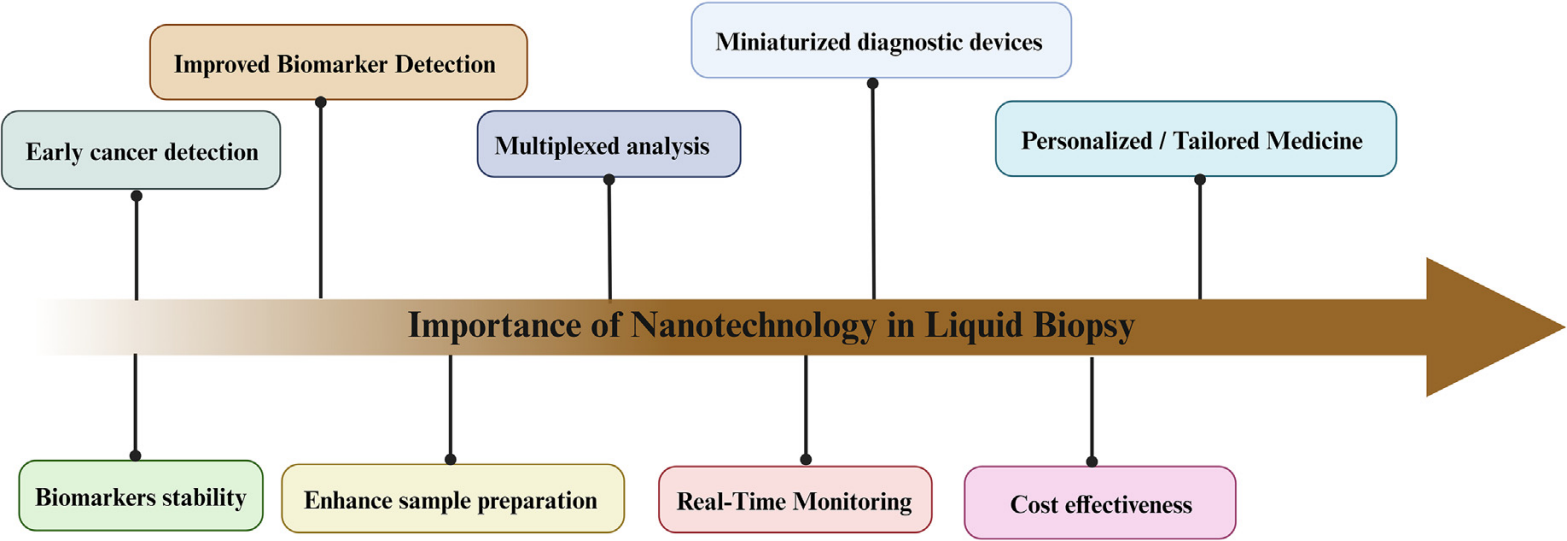
**Importance of nanobiotechnology in liquid biopsy** (Created with Biorender.com).

Biosensor based strategies holds great potential for transforming the clinical management of solid tumor because of following reasons [[27], [28], [29], [30], [31]]:

• Allows early detection and diagnosis as it specializes in detecting specific biomarkers associated with solid tumor. This allows early diagnosis and intervention thus contributing to significant improvement in treatment outcomes.
• Real-time monitoring of biomarker levels during the course of treatment thus contributing in assessment of the effectiveness of treatment.
• Biosensor based processes make biomarker monitoring process less invasive.
• It enhances the ease of performing Point-of-Care Testing.
• Biosensors may play important role in monitoring minimal residual disease helping in detection and quantification of traces of cancer cells that may remain after treatment.

Biosensor based cancer detection approaches may be applied in diverse clinical settings like cancer screening programs, diagnostic imaging processes, oncology clinics and oncology specialty hospitals, real-time monitoring during surgery, pathological laboratories, primary care and screening facilities, pediatric oncology, clinical trials and research settings and many more. Integration of nanobiosensor technologies with conventional healthcare strategies for improvement of screening, detection, diagnosis, treatment, and management of clinical cases is the fundamental objective of nanobiosensor based cancer detection techniques.

Studies have shown that the implementation of nanobiosensor based cancer biomarker detection strategies enhances the specificity and sensitivity of analytical processes, real-time monitoring of cancer biomarkers, efficient monitoring of minimal residual disease, comprehensive characterization of cancer biomarkers, minimizing the rate of false positives and negatives. Implementing nanobiosensor based cancer biomarker detection strategies have been shown to enhance the precision, and reliability of concerned processes [[25], [26], [27], [28], [29], [30], [31], [32]]. While nanotechnology has shown great promise in a variety of biomedical applications, including liquid biopsy, several technical and clinical limitations remain. Highly sensitive and specific biomarkers are essential for accuracy of liquid biopsy as the existing non-specific or low affinity nanomaterials tends to provide false positive or false negative results with a possible risk of toxicity. Understanding long-term impacts of nanoparticles are important for ensuring its biocompatibility before introduction into biological systems and strategizing clinical. The reproducibility of results and translation of nanotechnology based liquid biopsies into clinical practice across different laboratories are platforms are major concern because of differences in nanoparticle synthesis, functionalization, and assay protocols, standardization of nanotechnology-based assays. Nanomaterials used in liquid biopsy are expensive, limiting their widespread adoption. It is critical to develop scalable and cost-effective manufacturing methods for these nanomaterials so that liquid biopsy technologies can be made available to a larger population. Nanomaterials' stability may change over time, affecting their performance in applied liquid biopsy. Aggregation, degradation, or changes in surface properties could all contribute to stability issues. The efficiency with which nanomaterials capture and isolate target biomolecules, such as CTCs or cell-free nucleic acids, varies. Improving the efficiency of capture is critical for increasing the sensitivity of liquid biopsy methods. Liquid biopsy frequently involves the simultaneous detection of multiple biomarkers. Due to issues like cross-reactivity and interference between different detection elements, achieving multiplexing with nanotechnology can be difficult. Many nanotechnology-based liquid biopsy methods, while promising in laboratory settings, are still in the initial phases of development. To establish their reliability and accuracy in real-world patient populations, comprehensive clinical validation, including large-scale clinical trials, are essential. Liquid biopsy methods based on nanotechnology may necessitate sophisticated sample handling procedures. Keeping liquid biopsy samples stable and intact during collection, transportation, and processing is important for obtaining reliable results. Meeting regulatory requirements for approval and clinical use of nanotechnology-based liquid biopsy technologies are highly challenging. Getting past these regulatory roadblocks is critical for turning promising research into clinically viable diagnostic tools [[28], [29], [30]]. Nanotechnology become a frontier of the new Liquid Biopsy Era.

## Availability of data and materials

Data sharing does not apply to this article as no datasets were generated or analyzed during the current study.

## Funding

There is no funding for this study.

## Declaration of competing interest

The authors declare that they have no known competing financial interests or personal relationships that could have appeared to influence the work reported in this paper.
